# Improved Interobserver Reliability in Diagnosing and Staging Lesions of COVID-19 Between Radiologist and Emergency Medicine Physicians After an Online Course

**DOI:** 10.7759/cureus.29216

**Published:** 2022-09-15

**Authors:** Ashima Sharma, Madhuri Howdekar, Sarat Chandra U, Nithya Shreya Meghna, Abhishek J Arora

**Affiliations:** 1 Department of Emergency Medicine, Nizam's Institute of Medical Sciences, Hyderabad, IND; 2 Department of Radiology and Imgeology, Nizam's Institute of Medical Sciences, Hyderabad, IND; 3 Department of Emergency Medicine, Nizam's Institute Of Medical Sciences, Hyderabad, IND; 4 Department of Radiodiagnosis, All India Institute of Medical Sciences, Bibinagar, Hyderabad, IND

**Keywords:** chest infection, radiographic interpretation, covid 19, emergency medicine physician, diagnostic radiologist

## Abstract

Background: Chest radiographs are the most basic and readily available imaging modality for visualizing the lungs and are potentially useful for describing the disease severity in patients showing respiratory symptoms in COVID-19 patients. The early diagnosis of COVID-19 features on radiography helps in triaging and starting treatment.

Material and methods: Our study consisted of 145 radiographs, and these were reported by two radiologists, two emergency physicians and one intern working in the Emergency department. The scores given by them were correlated. A targeted short lecture for the scoring was imparted and after a sufficient latent period the scoring of chest radiographs was done again, and the scores correlated and compared.

Results: We observed agreement between radiologists with emergency medicine physicians was “none to slight” to “fair,” before the dedicated online teaching course. Following the meeting, there was an increase in interobserver agreement in-between radiologists and between radiologists and emergency medicine physicians.

Conclusions: We propose a focused online meeting, targeted at explaining radiological features of a specific pathology, in a pandemic situation like COVID, to our clinical counterparts in the emergency medicine department can help in increasing their interpretation skills. This can directly benefit triaging, admission/discharge and monitoring of the status of patients, in intensive care units and emergency medicine. This also helps in allaying the anxiety, while waiting for a final report from the Radiologist.

## Introduction

The Coronavirus Disease 2019 (COVID-19) is caused by a novel coronavirus called SARS-CoV-2, according to the Centers for Disease Control and Prevention, and primarily affects the lungs. WHO first learned of this new virus on December 31, 2019, following a report of a cluster of cases of “viral pneumonia” in Wuhan, People's Republic of China, and later declared COVID-19 as a public health emergency of international concern [1-3]. Chest radiographs are the most basic and readily available imaging modality for visualizing the lungs and are potentially helpful in describing the disease severity in patients showing respiratory symptoms in our institute. Due to the increased number of radiographs being ordered these days, for constant monitoring of the disease, they are usually assessed by an emergency medicine physician rather than a radiologist for further medical management is decided. The early diagnosis of COVID-19 is a critical task to start early treatment [4]. Our study assesses whether simple basic training by a radiologist to emergency medicine physician increases the accuracy of reporting of chest radiographs by emergency medicine physicians and if it increases the correlation between radiologists and emergency medicine physician reporting.

## Materials and methods

Our study included 145 radiographs of patients, who presented to the Emergency Department with COVID19 symptoms as defined by WHO, and for whom radiographs were taken as a screening tool. According to WHO, the case definition of severe acute respiratory illness consists of a patient with a history of fever or measured fever of ≥ 38 C°; and cough; with onset within the last 10 days, which requires hospitalization.

**Figure 1:**
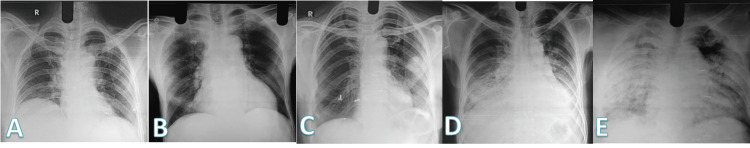
Scores given on chest radiographs. (A) Normal chest radiograph was given a score of 1, (B) radiograph with either patchy atelectasis as seen in the right lower zone was given a score of 2, (C) radiograph showing focal consolidation in the left midzone and given a score of 3, (D) chest radiograph showing multifocal consolidation in bilateral lower zones, silhouetting the left hemidiaphragm is given a score of 4, (E) diffuse consolidation seen in both lungs are given a score of 5.

Following the literature review, we used a five-point chest radiograph scoring tool to record the severity of lung abnormalities, with scores 1 being normal, 2for patchy atelectasis and/or hyperinflation or bronchial wall thickening. In addition, score 3 is given for focal consolidation, 4 for multifocal consolidation, and 5 for diffuse alveolar consolidation, as explained in Figures 1A-1E [5]. The given reference article was read by all the readers, before scoring the chest radiographs.

Total of five physicians: two radiologists, a senior radiologist with 15 years of experience and a junior radiology resident; two emergency medicine physicians consisting of a senior emergency medicine physician with 20 years of experience, a junior emergency medicine resident, and one medical intern working in the emergency department were included to assess chest radiographs of patients included in the study. These five readers independently and blindly reviewed all chest radiographs, and these radiographs were assigned a score of 1 to 5 for each radiograph. After completion of this exercise, the scores of all the study participants were tabulated, and we assessed Interobserver agreement with weighted kappa (κ) score [6].

Later, a gap of eight weeks was given, to prevent reporting recall bias. An online meeting and learning class were convened, and the senior radiologist explained in detail the radiographic features of consolidation and the imaging features of radiographs for scoring. After the discussion and due clarification of all the doubts of assessing participants, they were given the same radiographs, randomly, to evaluate and score as per the previous scoring system. Tabulating each score and analyzing it was repeated, and κ scores were assessed.

We compared the scores and determined the agreement between a pair of radiologists, emergency medicine physicians, and a medical intern, each with every other participant. The study score of senior radiologists was used as the reference standard. Weighted Kappa scores are defined as - κ < 0; No agreement, κ 0.01-0.20; None to slight, κ 0.21-0.40; Fair, κ 0.41-0.60; Moderate, κ 0.61-0.80; Substantial and, κ 0.81-1.00; Almost perfect agreement.

## Results

During the initial exercise, weighted kappa scores between the senior radiologist and radiology resident were fair (κ = 0.215). The κ values for agreement between emergency medicine physicians with radiologists ranged from 0.18 to 0.30, from “none to slight” to “fair.” Agreement of the senior radiologist scoring with the emergency medicine physician (κ = 0.21), emergency medicine resident (κ = 0.28) and medical intern (κ = 0.30) were all in the range of “fair” correlation. Agreement of the radiology resident's scoring of the CXR reports with the senior emergency medicine physician was none to slight (κ = 0.18), emergency medicine resident was none to slight (κ = 0.19); while with the medical intern, it was fair (κ = 0.22).

**Figure 2:**
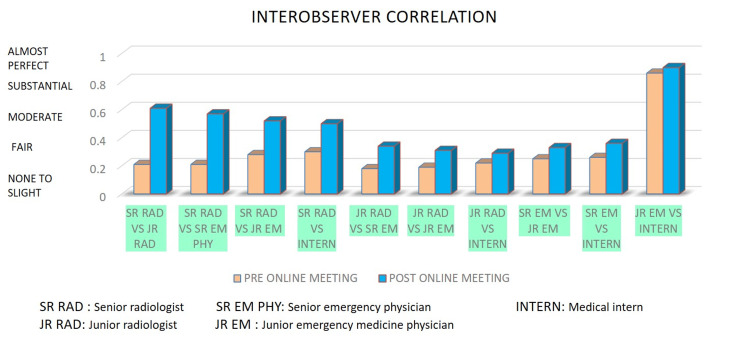
Graph showing interobserver correlation in-between senior and junior radiologists and between radiologists and emergency medicine physicians, before and after an online course.

The κ values for agreement between clinician pairs ranged from 0.25 to 0.86 “fair” to “almost perfect agreement.” For example, the agreement between the senior emergency medicine physician and emergency medicine resident was fair (κ = 0.25) versus the medical intern was fair (κ = 0.26), and the emergency medicine resident versus the medical intern was almost perfect agreement (κ = 0.86). These scores, as shown in Figure 2, clearly show that emergency medicine physicians depicted the lucencies and opacities over the radiographs similarly, with the almost perfect agreement between the junior doctors.

Chest radiograph scoring system following an online meeting

The κ values for agreement between the senior radiologist and radiology resident were substantial (κ = 0.61). Also, the κ values for clinicians' agreement with radiologists increased from 0.29 to 0.90 “fair” to “almost perfect agreement.” Agreement of the senior radiologist scoring of the reports with the senior emergency medicine physician (κ = 0.57), emergency medicine resident (κ = 0.52), medical intern (κ = 0.50) was moderate while, agreement of the radiology resident with the senior emergency medicine physician (κ = 0.34), emergency medicine resident (κ = 0.31) and with medical intern (κ = 0.29) was in “fair” category. In all three instances, between two radiologists, senior radiologists and emergency physicians, and junior radiologists and emergency physicians, the k-weighted scores increased substantially.

The κ values for agreement between clinician pairs ranged from 0.33 to 0.90. The agreement between the senior emergency medicine physician and emergency medicine resident was “fair” (κ = 0.33) versus the medical intern (κ = 0.36), and the emergency medicine resident versus the medical intern was almost perfect agreement (κ = 0.90).

## Discussion

Using a novel five-point ordinal scoring system, we described the agreement of emergency medicine physicians with radiologists and in-between emergency medicine physicians in interpreting chest x-ray (CXR) abnormalities in patients with symptoms of COVID-19. We observed “fair” interobserver agreement between radiologists. Agreement between radiologists with emergency medicine physicians was “none to slight” to “fair.” Interobserver agreement between clinicians of various experience levels was “fair” to “almost perfect.” The above interobserver agreements highlighted that the interpretation skills of non-radiologists are similar in many ways. They interpret various opacities over the chest radiograph in a similar fashion reaching similar conclusions and high correlation scores between them.

Following an online meeting, there was a “substantial” increase in interobserver agreement between radiologists who reviewed the radiographs and applied the scoring system. Agreement between radiologists with clinicians was “fair” to “almost perfect” understanding, while Interobserver agreement between clinicians of various levels of experience was “fair” to “almost perfect.” Post online teaching, there was a substantial increase in the Interobserver agreement between two radiologists and between radiologists and emergency physicians. There was not much increase in correlation between the non-radiologists, as it was already near perfect before they attended online score. However, the takeaway message was there was neither a decrease in the correlation, suggesting both the junior emergency physician and the medical intern improved their skills of interpretation of radiographs, improving the correlation scores with radiologist counterparts.

On reviewing the literature, very few studies online compare the Interobserver correlation. Cristian et al. have done chest x-ray severity scores in patients with reverse transcriptase-polymerase chain reaction positive for SARS-CoV-2 and CXR images on ED admission and proposed, CXR pulmonary severity score in COVID-19 showed moderate to an almost perfect interobserver agreement and significant but weak correlations with clinical parameters, potentially furthering CXR integration in patients' stratification [7].

Borghesi et al. retrospectively evaluated correlations between the CXR score and the age or sex of Italian patients infected with SARS-CoV-2, and their results showed males aged 50 years or older and females aged 80 years or older showed the highest risk of developing severe lung disease and concluded their results might help to identify the highest-risk patients and those, who require specific treatment strategies [8].

During pandemics and large epidemiological surveillance projects, chest radiographs have been used extensively as a screening tool. They are standard of care and help maintain objectivity in all parts of the world and thus help monitor the progression and follow-up of the disease, especially in intensive care units. Due to the increasing burden of the disease, our study validates that by focused, targeted teaching of the emergency medicine physicians about a specific pathology, a good correlation between them and radiologists in interpreting the chest radiographs. This forms a basis for better communication of findings and independent and faster decision-making at the time of admission and start of treatment and course correction during treatment of patients.

## Conclusions

Our study proves that the interobserver correlation between the radiologist and emergency medicine physicians improved significantly after a focused online meeting targeted at explaining a specific pathology. These types of short, targeted lectures, help in teaching specific imaging findings to the clinicians for a particular disease and thus increase their confidence in interpreting those conditions, till the final report by a trained Radiologist reaches them. A simple five-point ordinal scoring system of chest radiographs can be used as a valuable and reliable tool in assessing the severity of lung consolidation due to any cause, by emergency medicine physicians of various levels of experience and it leads to better communication of findings and stratification of severity of the disease.

We propose a focused online meeting, targeted at explaining radiological features of a specific pathology, in a pandemic situation like COVID, to our clinical counterparts in the emergency medicine department can help in increasing their interpretation skills. This can directly benefit triaging, admission/discharge, and monitoring of the status of patients, in intensive care units and emergency medicine. This also helps in allaying the anxiety, while waiting for a final report from the Radiologist.
